# Therapy Mode Preference Scale: Preliminary Validation Methodological Design

**DOI:** 10.2196/65477

**Published:** 2024-11-29

**Authors:** Brianna Cerrito, Jamie Xiao, Amanda Fialk, Frank D Buono

**Affiliations:** 1 The Dorm, New York, NY, United States; 2 Yale School of Medicine, New Haven, CT, United States

**Keywords:** virtual teletherapy, young adult mental health treatment, in-person therapy, virtual mental health care, telehealth, mental health treatment, virtual care, therapeutic, virtual therapy, in-person treatment, exploratory factor analysis, hierarchical linear regression, standardized tool, herapeutic impact

## Abstract

**Background:**

The use of tele–mental health care increased rapidly in 2020 as a critical response to the COVID-19 pandemic, serving as an effective contact-free alternative to treatment. Today, tele–mental health care remains a viable option for individuals with geographic and physical barriers to treatment. However, there are several potential therapeutic disadvantages to tele–mental health care (ie, missing nonverbal signals, handling crises, confidentiality, weakened social connection in group therapy) that should be evaluated. While published literature has explored client satisfaction within teletherapy and the effect of using technology for tele–mental health care demands, there is a need for published surveys that evaluate the therapeutic experience in teletherapy and in-person mediums of care.

**Objective:**

The authors of this study sought to develop and validate a survey that could evaluate the comparative impact of teletherapy and in-person care from a therapeutic perspective across key factors (ie, therapeutic alliance, engagement, rapport, and confidentiality).

**Methods:**

Participants were clients who experienced both tele–mental health care and in-person therapy at an intensive outpatient mental health treatment program for young adults from April 2020 through June 2022. Generated items on the survey were formulated based on input from experts in the field and existing validated scales. All individuals completed the survey on the internet, following informed consent (n=89). An exploratory factor analysis was conducted to understand factor structure, and Cronbach α was used to determine internal consistency. Incremental validity was demonstrated through a hierarchical linear regression.

**Results:**

The exploratory factor analysis revealed a 14-item, 3-factor structure. All 14 items correlated at a minimum of 0.30 with at least one other item. Kaiser-Meyer-Olkin measure of sampling adequacy was 0.75 and Bartlett’s test of sphericity was significant (*χ*^2^_91_=528.41, *P*<.001). In total, 3 factors accounted for 61% of the variance, and the preliminary Cronbach α (α=0.71) indicates a satisfactory level of internal consistency. The Zoom Exhaustion and Fatigue Scale (ZEF) and Client Satisfaction Questionnaire (CSQ; −0.29) were significantly correlated, as well as the ZEF and Therapy Mode Preference Scale (TMPS; −0.31), and CSQ and TMPS (0.50; *P*<.001). Hierarchical linear regression revealed that the CSQ significantly accounted for additional variance in the TMPS (*P*<.001). With the ZEF entered into the model, no further variance was accounted for (*P*=.06).

**Conclusions:**

Continual research is warranted to expand the current findings by validating this standardized tool for assessing the therapeutic impact of teletherapy versus in-person care in a generalizable population.

## Introduction

The use of tele–mental health care increased rapidly in 2020 as a critical response to the COVID-19 pandemic, effectively serving as a contact-free alternative to receiving treatment [1-3]. Tele–mental health care remains a viable option for individuals with geographic and physical barriers to treatment, and recent research has demonstrated that teletherapy can be as effective as in-person sessions for treatment outcomes [1,4,5]. However, there are also several perceived therapeutic disadvantages to teletherapy (ie, missing nonverbal signals, handling crises, confidentiality, weakened social connection in group therapy) [2,3,6-8]. These disadvantages can affect the relationship between clinician and client, which has been proven to be a key factor in treatment outcomes [9-12].

Web-based therapy settings can provide a convenient space for clients, but they also offer a set of disadvantages in maintaining the therapeutic relationship. A recent study examining the impact of tele–mental health care on clients enrolled in chronic pain therapy reports that while teletherapy sessions may provide a more comfortable setting for the client, tele–mental health treatment is also associated with artificial dialogue and superficial connection, lower levels of empathy, and greater self-consciousness [13]. Additionally, clinicians and clients who have experienced in-person therapy report that the personal aspects of therapy (ie, connection, vocal nuances, nonverbal communication, and body language) were missing from teletherapy, making it overall less effective [14]. Other disadvantages to teletherapy include being unable to pick up on nonverbal cues, issues with confidentiality, and fatigue associated with technology use [6,8,15]. Though teletherapy can be useful in providing access to those who may be unable to attend in person, the drawbacks to these therapy sessions are also clinically significant factors that should be considered.

Current research lacks validated scales that comprehensively measure the effects of teletherapy versus in-person mediums of therapy on the therapeutic experience. Weaver et al’s [15] review of telehealth scales explains that many of the existing instruments examine the perceived quality of communication and technology interface but not specific therapeutic effects. For instance, the Telemedicine Satisfaction and Usefulness Questionnaire only measures patient satisfaction with telehealth services, while the Zoom Exhaustion and Fatigue Scale (ZEF Scale) measures only the effect of using technology for a prolonged period [16,17]. The former scale evaluates satisfaction, which does not account for the therapeutic experience and alliance that often drive outcomes, and while the latter does address the effect of technology, its focus is on fatigue rather than therapeutic impact. Other scales like this, such as the Telehealth Usability Questionnaire, which evaluates the usability of telehealth implementation and services, are useful for better understanding the adaptation of tele–mental health care but not therapeutic impact [15,18].

While published literature has explored client satisfaction within teletherapy and the effect of using technology for tele–mental health care demands, there is a clear gap in research examining validated surveys that compare therapeutic impact of teletherapy and in-person mediums of care [15-18]. Thus, the authors of this study sought to develop a survey that could evaluate the comparative impact of teletherapy and in-person care from a therapeutic perspective.

To establish initial construct validity, we hypothesized that a factor analysis would identify 3 subscales (ie, therapeutic factors in virtual care, therapeutic factors in in-person care, and general factors in virtual care). We also hypothesized that the Therapy Mode Preference Scale (TMPS) desirability subscales would be significantly positively associated with existing tele–mental health care metric outcomes and explain unique additional variance above and beyond endorsement ratings. Our third hypothesis was that TMPS subscales would explain variance in outcomes related to tele–mental health care above and beyond existing measures of satisfaction and fatigue in web-based treatment.

## Methods

### Procedures

#### Item Generation

Item generation and initial questionnaire development were conducted for the Therapy Mode Preference Scale (TMPS) through a mixed methods approach that included a review of the existing literature, input from seasoned clinicians, and previously validated scales on the topic.

A comprehensive literature review was conducted, encompassing relevant studies on teletherapy, the therapeutic relationship, and peer connection. The review highlighted recurring themes such as those related to technology (ie, technical difficulties, confidentiality concerns) and rapport factors (ie, connectedness and comfort in the therapeutic and peer relationships), underscoring the need for a questionnaire that addresses general factors in tele–mental health care as well as specific rapport and therapeutic alliance questions in teletherapy versus in-person settings.

Clinician consultation involved 3 expert clinicians in social work who were selected based on their extensive experience working with a multi-diagnostic young adult population in both teletherapy and in-person settings. During feedback sessions, clinicians provided valuable insights into key aspects of therapy that are important for treatment progress, informing the item generation process as well as selection and refinement of questionnaire items. At each session, conversations were recorded with each clinician, followed by transcription of comments. Results were analyzed to determine the most effective and essential aspects of therapy that would be necessary to include in the TMPS. Content deemed critically important was used for the first round of item generation.

Following expert feedback, items were further adapted and generated based on input from a selection of validated scales related to tele–mental health care. The first survey that questions were adapted from is the Client Satisfaction and Experience with Telepsychiatry Survey [19]. This is a 20-item survey that assesses satisfaction with telepsychiatry on a 5-point Likert scale (1=strongly disagree, 5=strongly agree). Items were also adapted from the Group Engagement Measure, a 27-item survey that assesses engagement in group therapy across 7 subscales (ie, attending, contributing, relating to workers, relating with members, contracting, working on one’s own problems, working with others’ problems) on a 5-point Likert scale (1=rarely or none of the time, 5=most or all of the time) [20].

Generated items were formulated based on input from clinicians, while other adapted items were based on the content of established scales (Textbox 1).

Following iterative cycles of brainstorming and refinement, the result was a selection of 3 factors: general factors in virtual care (ie, confidentiality, privacy, audio issues), therapeutic factors in virtual care, and therapeutic factors in in-person care.

Textbox 1. **15-Item therapy mode preference scale, designed to evaluate virtual and in-person mental health treatment.**

Directions: In your overall experience receiving virtual treatment at the Dorm, please rate the statements based on how true they are to you. Please be honest in your responses and review the ranking system below before proceeding.

1=Very Untrue; 2=Untrue; 3=Neutral; 4=True; 5=Very True

**General**

1. I believe virtual sessions at the Dorm are just as effective as in-person sessions. [19].

2. I was able to see my clinicians clearly in virtual sessions. [19]

3. I was able to hear my clinicians clearly in virtual sessions. [19]

4. I experienced technical difficulties that impacted my experience with virtual care.

5. My confidentiality was protected in virtual sessions. [19]

**Virtual. Please answer the following questions regarding virtual care.**

6 I changed because of virtual sessions. [19]

7. My clinician was more approachable in virtual sessions.

8. I was able to connect with my therapist more in virtual sessions.

9. I was able to maintain relations with other clients in virtual sessions. [20]

10. I was more comfortable sharing feelings in virtual sessions. [19]

**In-person. Please answer the following questions regarding in-person care.**

11. I changed because of in-person sessions. [19]

12. My clinician was more approachable in in-person sessions.

13. I was able to connect with my therapist more in in-person sessions.

14. I was able to maintain relations with other clients in in-person sessions. [20]

15. I was more comfortable sharing feelings in in-person sessions. [19]

#### Recruitment

The research manager and research assistants contacted a sample of clients who experienced both teletherapy and in-person therapy at an intensive outpatient mental health treatment program for young adults, The Dorm, from April 2020 through June 2022. Individuals were invited to participate in the study via email. Inclusion criteria were (1) active clients at The Dorm between April 2020 and June 2022, (2) provided informed consent, and (3) 18 years or older. Exclusion criteria were (1) unfit to complete the survey due to medical or psychological constraint, (2) not fluent in the English language, and (3) unwilling to complete the entire survey. Individuals who did not qualify were not given the link to the survey. Individuals who did qualify for the study were sent a link for the Qualtrics survey.

### Measures

The TMPS is a 15-item self-report assessment that evaluates tele–mental health care and in-person care across key therapeutic and technical domains. This survey contains 3 subscales (general factors in virtual care, the therapeutic impact of virtual care, and the therapeutic impact of in-person care) with 5 items in each scale. The responses are rated on a 5-point Likert scale (1=very untrue, 2=untrue, 3=neutral, 4=true, 5=very untrue), taking on average 7.5 minutes.

The CSQ-8 is an 8-item survey used to assess satisfaction of client experience (α=0.93) [21]. Given that the root of each item varies (ie, “How would you rate the quality of service you received?”; “How satisfied are you with the amount of help you received?”), the anchors vary across each of the 8 items. All items are rated on a 4-point scale [22].

The ZEF Scale is a 15-item scale measuring 5 aspects of fatigue experienced in Zoom videoconferences, which include general (α=0.90), visual (α=0.88), social (α=0.87), motivational (α=0.93), and emotional (α=0.93) [16]. All items are measured on a 5-point Likert scale (1=not at all, 2=slightly, 3=moderately, 4=very, 5=extremely) except for 2 frequency items (1=never, 2=rarely, 3=sometimes, 4=often, 5=always).

### Data Collection

#### Location

The Dorm is an intensive outpatient program for young adults, ages 18‐35 years, located in New York City and Washington, DC. Program duration is approximately 1 year, on average. Treatment includes (1) empirically supported behavioral psychosocial methodologies to serve a variance of mental health illnesses and co-occurring disorders; (2) alternative and complementary modalities (ie, exercise, yoga, reiki, horticulture, community service, meditation, mindfulness); (3) family programming including weekly parent coaching, parent groups and family groups; (4) clients work with both a therapist and a clinical coach; and (5) participation in 3‐30 hours a week of group therapy, depending on the treatment phase.

#### Administration

Surveys were administered via email. Recruitment emails were sent to clients who had sought intensive outpatient treatment who were actively seeking treatment at The Dorm between April 2020 and June 2022. All individuals were provided with an ID number for confidentiality, as all surveys were completed on the web. Informed consent was obtained via e-signature prior to survey completion. Using the Qualtrics survey platform, clients filled out the TMPS and 2 additional surveys for the purpose of assessing concurrent validity (CSQ-8, ZEF) [16,21].

### Ethical Considerations

This study is approved by the Institutional Review Board at Yale School of Medicine (IRB #2000032626). Informed consent was collected in Qualtrics via e-signature before completing the survey. The consent form ensured participants that the study data are confidential, as data are linked to a deidentified code instead of client names. Data are protected in an encrypted Qualtrics server, and the master sheet that matches client names to their codes is also stored in an IT-protected Google server. Clients who participated were entered into a lottery to win a US $100 Amazon gift card.

### Data Analytic Strategy

Exploratory factor analysis (EFA) was conducted on the TMPS using SPSS (IBM Corp). EFA on the initial 15 items was conducted using principal component analysis with Varimax rotation (Kaiser normalization). In total, 3 factors were retained, as the survey was designed to have 3 sections, each measuring different aspects of mental health care by medium. Items were retained if they had a primary factor loading greater than 0.30 and did not cross-load onto multiple factors. Items that had factor loadings less than 0.30 were removed from the scale. EFA was repeated using principal component analysis with Varimax rotation to confirm factor structure results if items were removed. Once a simple structure was achieved, a reliability analysis was conducted for each retained factor (3), excluding the eliminated items from the analysis. Properties of the TMPS were tested among the full sample.

First, the internal consistency was examined using Cronbach α. Second, concurrent validity was tested using bivariate correlations with 2 outcome variables: ZEF and CSQ. Last, incremental validity of the TMPS was via a series of hierarchical linear regression models. CSQ was entered at step 1, and ZEF was entered at step 2. Prior to analysis, correlations were examined to ensure multicollinearity was within normal limits for running the analysis.

## Results

### Overview

A total of 439 individuals were initially recruited for the study. In total, 118 individuals opted in to complete the study, but 29 participants did not fully complete it, thus failing to meet inclusion and exclusion criteria. A total of 89 participants met criteria to be included in the analysis.

Participants were young adults between the ages of 18 and 34 (mean 23.64, SD 0.37) years seeking treatment at an intensive outpatient mental health treatment program in New York, NY and Washington, DC (Table 1). Of the 90 clients in the study, 37% (n=33) identify as cisgender female, 38% (n=34) as cisgender male, 16% (n=14) as transgender nonbinary, 4% (n=4)unknown or exploring, and 5% (n=5) did not list gender. The majority of the clients were students (n=36, 40%) and had a history of trauma (n=68, 76%). Some clients reported having been diagnosed with a substance use disorder (n=38, 31%). When asked about frequency of teletherapy sessions per week, most of the clients reported attending 1‐5 teletherapy sessions per week (n=75, 83%), with fewer clients reporting 6‐10 sessions per week (n=10, 11%), 11‐15 sessions per week (n=1, 1%), 16‐20 sessions per week (n=3, 3%), and ≥21 sessions per week (n=1, 1%).

**Table 1.** Demographics table of admission intakes (gender, employment status, history of trauma, substance use diagnosis, and frequency of virtual sessions).

Demographic variables	Participants, n (%)	
Gender		
Cisgender female	33 (37)	
Cisgender male	34 (38)	
Transgender nonbinary	14 (16)	
Unknown or exploring	4 (4)	
Did not disclose gender	5 (5)	
Employment status		
Employed	30 (33)	
Student	36 (40)	
Unemployed	24 (27)	
History of trauma		
Yes	68 (76)	
No	22 (24)	
Substance use diagnosis		
Yes	28 (31)	
No	62 (69)	
Frequency of virtual sessions per week		
1‐5	75 (83)	
6‐10	10 (11)	
11‐15	1 (1)	
16‐20	3 (3)	
≥21	1 (1)	

### Exploratory Factor Analysis

Of the 15 items, 14 were above the 0.30 minimum threshold. In total, 1 item (question 8) was excluded from the analysis as it loaded on 2 factors in the principal component analysis. A 3-factor EFA was used to examine the factorability of 14 items in the TMPS. The data were screened for univariate outliers, and the minimum amount of data for factor analysis was satisfied. The factorability of the 14-item TMPS was examined using several well-recognized criteria. All 14 items correlated at a minimum of 0.30 with at least one other item. Kaiser-Meyer-Olkin measure of sampling adequacy was 0.75, above the recommended value of 0.50 [23]. Bartlett’s test of sphericity was significant (*χ*^2^_91_=528.41; *P*<.001). To determine the number of factors to extract, a scree plot of the eigenvalues was examined using the elbow criterion, where the tapering of the plot clearly indicated a unidimensional structure [24].

Diagonals of the anti-image correlation matrix exceeded 0.30, supporting inclusion of each item in the factor analysis. Communalities ranged between 0.37 and 0.80, confirming all other items shared some common variance. Principal component analysis with varimax rotation was used because the primary purpose was to identify and compute scores for the correlated factors underlying the TMPS (Table 2). In total, 3 factors accounted for 61% of the variance. The first factor explained 25% of the variance, the second factor explained 18% of the variance, and the third factor explained 18% of the variance. A varimax rotation provided the best-defined factor structure. Preliminary Cronbach α (α=0.71) indicates a satisfactory level of internal consistency for our measurement instrument.

**Table 2.** Factor matrix for a 3-factor model of a 14-item survey evaluating general factors in virtual care, the therapeutic impact of in-person care, and the therapeutic impact of virtual care.

Survey items	Factor 1	Factor 2	Factor 3
I believe virtual sessions at The Dorm are just as effective as in-person sessions	−0.14	0.23	0.78
I was able to see my clinicians clearly in virtual sessions	0.05	0.80	0.18
I was able to hear my clinicians clearly in virtual sessions.	0.09	0.87	0.20
I experienced technical difficulties that impacted my experience with virtual care.	0.04	0.73	−0.13
My confidentiality was protected in virtual sessions.	0.17	0.65	0.04
I changed because of virtual sessions.	0.25	0.01	0.64
My clinician was more approachable in virtual sessions.	−0.14	−0.19	0.74
I was able to maintain relations with other clients in virtual sessions.	−0.05	0.13	0.59
I was more comfortable sharing feelings in virtual sessions.	−0.28	0.08	0.68
I changed because of in-person sessions.	0.67	0.23	0.10
My clinician was more approachable in virtual in-person sessions.	0.86	−0.02	−0.12
I was able to connect with my therapist more in in-person sessions.	0.86	0.07	−0.15
I was able to maintain relations with other clients in in-person sessions.	0.79	0.21	−0.08
I was more comfortable sharing feelings in in-person sessions.	0.82	−0.03	−0.11

### Concurrent Validity

Correlation coefficients were evaluated for each of the scales used (Table 3) ZEF and CSQ (−0.29), ZEF and TMPS (−0.31), and CSQ and TMPS (0.50) were significantly correlated (*P*<.0o1). Higher CSQ scores indicate higher satisfaction, whereas higher ZEF scores indicate more extreme Zoom fatigue.

**Table 3.** Correlation matrix examining the relationship between the Client Satisfaction Questionnaire (CSQ), Zoom Exhaustion and Fatigue Scale (ZEF), and Therapy Mode Preference Scale (TMPS).

	CSQ	ZEF	TMPS
CSQ			
*R*	1	–0.29	0.50
*P* value	—^a^	<.001	<.001
ZEF			
*R*	−0.29	1	–0.31
*P* value	<.001	—	<.001
TMPS			
*R*	0.50	–0.31	1
*P* value	<.001	<.001	—

a Not applicable.

### Incremental Validity

The hierarchical linear regression revealed a highly significant relationship between the CSQ and TMPS. Incremental validity is significant; it is only with the CSQ. A hierarchical linear regression was conducted for the TMPS with CSQ and ZEF scales entering the model. The coefficient for CSQ was significant (*P*<.001) but not for ZEF (*P*=.06), indicating that only CSQ accounted for additional variance in the TMPS.

Multicollinearity was within normal limits for all models. With TMPS and CSQ entered the model, CSQ significantly accounted for additional variance in the TMPS (*P*<.001) (Table 4). With the ZEF entered the model, no additional variance was accounted for (*P*=.06).

**Table 4.** Hierarchical linear regression predicting Therapy Mode Preference Scale scores with Client Satisfaction Questionnaire (CSQ) and Zoom Exhaustion and Fatigue Scale (ZEF).

Predictors	Beta	Standard error	Standardized beta	*t* test (*df*)	*P* value
CSQ	0.04	0.01	0.45	4.69 (1)	<.001
ZEF	−0.10	0.05	−0.18	−1.88 (2)	.06

## Discussion

### Principal Findings

This study developed and examined the initial construct validity and psychometric properties of the TMPS. Results of the reliability analysis indicate that the TMPS has good reliability, and the EFA supported a 14-item 3-factor structure. It can be understood that the TMPS is valid as a measure of the following 3 factors: therapeutic impact of in-person care, general factors in virtual care (ie, technology), and therapeutic impact of virtual care. General factors in virtual care are concerning logistics and safety, such as technology (seeing clearly, hearing), confidentiality, and client perception of effectiveness, which contribute to the therapeutic process being possible. Logistics are important to evaluate from a feasibility and quality assurance standpoint, as being unable to see or hear properly can disrupt the therapeutic environment. Therapeutic impact of virtual and in-person care, however, pertains to feelings of approachability, comfort, connectedness, and ability to change, all of which are components that make therapy effective. For both teletherapy and in-person treatment, it is important to evaluate the presence of these facets of therapy, as these factors may contribute to client motivation, thus contributing to the likelihood of increasing adherence to treatment.

The authors found that ZEF, CSQ, and TMPS were all significantly correlated, indicating agreement between the 2 chosen assessments measuring the same construct, pointing to good concurrent validity. The CSQ significantly accounted for additional variance in the TMPS, whereas ZEF did not, suggesting that client satisfaction has predictive power over ratings of therapy in different mediums of care.

The therapeutic alliance in treatment is a critical component for the potential of successful outcomes; various meta-analyses have found a moderate but robust correlation between the quality of the therapeutic alliance and treatment outcomes [9,11,12]. Research shows that patients’ perception of patient-clinician interaction is one of the most important determinants of satisfaction within clinical service [25]. In addition, having a good rapport between therapist and client was found to be correlated with better outcomes and significant improvements in client well-being [10]. For this reason, the TMPS was designed to evaluate the therapeutic relationship between client and therapist, as impacted by teletherapy and in-person interactions. Existing assessments evaluate outcomes solely based on symptom reduction, rather than the therapeutic relationship.

Recent research reports that outcomes are not significantly different between in-person and telehealth treatment for decreasing depressive symptoms and increasing quality of life, as measured by the Quick Inventory of Depressive Symptomatology-Self-Report and Quality of Life Enjoyment and Satisfaction Questionnaire [26]. Some studies even indicate that treatment completion rate is higher for those in telehealth than those required to be in person [27]. However, following an extensive literature review, there is a gap in validated scales that can effectively measure the comparative impact of teletherapy and in-person therapy to ensure the best possible quality of care for clients. While teletherapy has several therapeutic benefits, it is also important to consider that there are therapeutic factors natural to in-person contact that may be weakened or negatively impacted within a web-based environment (ie, social connection, therapeutic alliance, and relationship-building) [2,3,6-8]. Previously validated assessments measuring tele–mental health care focus indirectly on therapeutic impact by measuring satisfaction of care received and topics related to social, emotional, and motivational aspects of web-based mediums (ie, Zoom videoconferencing) [16,21]. For this reason, the authors sought to develop a survey that focused specifically on the comparative therapeutic impact of medium of care to uncover how teletherapy care might differ from the effect of in-person therapy.

### Clinical Implications

There are important clinical and treatment implications of this study. While digital interventions indeed have benefits for rural communities and provide added conveniences (ie, lack of commuting or late-night options), in-person treatment may be preferred in certain situations (ie, high acuity mental health concerns). Moreover, understanding the impact of the therapeutic alliance between the therapist and the patient is critical. Increasing therapeutic alliance will increase the likelihood of a stronger emotional connection and goal-directed therapeutic environment in either a digital or in-person setting. In the utilization of the current assessment, therapists can deem an important intermediary understanding of the ideal treatment environment, which can directly affect the patient’s well-being.

Emerging evidence has demonstrated the feasibility of individuals with early psychosis in utilizing virtual group therapy [28]. Given these findings, the administration of the current assessment could be useful for future clinical trials. Researchers can use the current assessment to assess and control for baseline conditions and potential exclusionary criteria. In doing so, the future clinical trials would reduce the likelihood of potential errors and increase the treatment’s therapeutic effects.

### Limitations

There were several limitations in this study that should be considered in future research. First, clients were a specific set of individuals in intensive outpatient mental health treatment in New York, NY and Washington, DC. Due to the specificity of this sample, the findings may not be generalizable to other populations. It is important that future research seeks to validate this survey in diverse populations. Second, discriminant validity was not assessed in this study but is warranted in future research. Third, the sample size was low, and one item had to be dropped due to not loading with the other factors. Fourth, there was a lack of diversity in the study, as most clients were white individuals from high socioeconomic status. Fifth, acuity levels were not accounted for in this sample either; however, it is important to note that clients included in the study have varying acuity, which ranges from severe to mild. Finally, web-based survey administration does have limitations, such as socioeconomic differences in access to internet, which also impacts response bias (ie, research shows that respondents with higher education tend to give less truthful responses) [29]. The authors did their due diligence to use features within Qualtrics to minimize the possibility of bias.

### Conclusions

It is important to be able to evaluate the impact of web-based mediums on therapeutic factors in care. This study provides support for a potential evaluation tool that could be of use for mental health clinicians conducting hybrid or tele–mental health care using web-based conferencing. Based on the results of this study, preliminary psychometrics are favorable for a new survey designed to evaluate the comparative impact of teletherapy and in-person mental health treatment. The goal is for this assessment tool to be used in clinical settings to ensure therapeutic impact is as effective in a teletherapy setting as in-person. Continual research is warranted to establish a standardized tool for assessing the therapeutic impact of teletherapy versus in-person care.

## Abbreviations

CSQ: Client Satisfaction Questionnaire
EFA: exploratory factor analysis
TMPS: Therapy Mode Preference Scale
ZEF: Zoom Exhaustion and Fatigue Scale
